# Diagnosing Fungal Keratitis and Simultaneously Identifying *Fusarium* and *Aspergillus* Keratitis with a Dot Hybridization Array

**DOI:** 10.3390/jof8010064

**Published:** 2022-01-07

**Authors:** Ming-Tse Kuo, Shiuh-Liang Hsu, Huey-Ling You, Shu-Fang Kuo, Po-Chiung Fang, Hun-Ju Yu, Alexander Chen, Chia-Yi Tseng, Yu-Hsuan Lai, Jiunn-Liang Chen

**Affiliations:** 1 Department of Ophthalmology, Kaohsiung Chang Gung Memorial Hospital and Chang Gung University, College of Medicine, Kaohsiung City 83301, Taiwan; fangpc@cgmh.org.tw (P.-C.F.); angelayu@cgmh.org.tw (H.-J.Y.); a1050276@hotmail.com (A.C.); ppp6692001@cgmh.org.tw (C.-Y.T.); hurray@cgmh.org.tw (Y.-H.L.); 2 School of Medicine, Chang Gung University, Taoyuan City 33302, Taiwan; 3 Department of Ophthalmology, Chung-Ho Memorial Hospital, Kaohsiung Medical University, Kaohsiung City 80708, Taiwan; shiuhlianghsu@yahoo.com.tw; 4 Department of Laboratory Medicine, Kaohsiung Chang Gung Memorial Hospital and Chang Gung University, College of Medicine, Kaohsiung City 83301, Taiwan; youhling@cgmh.org.tw (H.-L.Y.); ivykuo@cgmh.org.tw (S.-F.K.); 5 Department of Medical Biotechnology and Laboratory Sciences, College of Medicine, Chang Gung University, Taoyuan City 333323, Taiwan; 6 Department of Ophthalmology, Kaohsiung Veterans General Hospital, Kaohsiung City 81362, Taiwan

**Keywords:** microbial keratitis, fungal keratitis, mycotic keratitis, keratomycosis, molecular diagnosis, *Fusarium*, *Aspergillus*

## Abstract

Fungal keratitis (FK) is one of the most common microbial keratitis, which often leads to poor prognosis as a result of delayed diagnosis. Several studies implied that early differentiation of the two major FK, *Fusarium* and *Aspergillus* keratitis, could be helpful in selecting effective anti-fungal regimens. Therefore, a novel dot hybridization array (DHA) was developed to diagnose FK and differentiate *Fusarium* and *Aspergillus* keratitis in this study. One hundred forty-six corneal scrapes obtained from one hundred forty-six subjects impressed with clinically suspected FK were used to evaluate the performance of the DHA. Among these patients, 107 (73.3%) patients had actual FK confirmed by culture and DNA sequencing. We found that the DHA had 93.5% sensitivity and 97.4% specificity in diagnosing FK. In addition, this array had 93.2% sensitivity and 93.8% specificity in diagnosing *Fusarium* keratitis, as well as 83.3% sensitivity and 100% specificity in diagnosing *Aspergillus* keratitis. Furthermore, it had 83.9% sensitivity and 100% specificity in identifying *Fusarium solani* keratitis. Thus, this newly developed DHA will be beneficial to earlier diagnosis, more precise treatment, and improve prognosis of FK, by minimizing medical refractory events and surgical needs.

## 1. Introduction

Fungal keratitis (FK) is an opportunistic corneal infection of fungi predisposed by corneal surface trauma [1]. According to the Asia Cornea Society Infectious Keratitis Study (ACS IKS) [2], FK was one of the most common microbial keratitis (MK), which was secondary to bacterial keratitis (BK) (FK:BK = 33%:38%). In addition, they found that trauma was the most common risk factor for MK. However, FK is easily overlooked due to its relatively sluggish progressive course. In addition, less intense pain in the early phase [3] often leads to longer delay before seeking medical care, which results in a worse visual outcome than BK. Previous reports showed clinical diagnosis of FK is highly challenging [4,5]. The sensitivity and specificity of clinical diagnosis of FK were 38% and 45%, respectively [4]. Even for an experienced corneal physician, the diagnostic accuracy via slit lamp image was only about 76% [6]. Consequently, about 12 to 58% of FK patients needed therapeutic keratoplasty or other surgeries to quiet down their infection episodes [7,8,9], and the surgical cure rate of FK was the worst among various MKs [10]. Finally, up to 25% of FK patients might lose their vision [11].

Among the 2831 isolated microorganisms in the ACS IKS [2], the top 3 pathogens were *Fusarium* spp. (18%), *Pseudomonas* spp. (10%), and *Aspergillus* spp. (8%). A recent comprehensive review confirmed *Fusarium* and *Aspergillus* as FK’s most common fungal isolates globally [11]. FK responds poorly to anti-fungal agents once the deep invasion of fungi occurs, which is why early diagnosis of FK is crucial. If diagnosis and treatment are made early, polyenes and azoles were active against *Fusarium* spp. and *Aspergillus* spp., respectively [12]. Moreover, the Mycotic Ulcer Treatment Trial (MUTT) also found that *Fusarium* spp. were least susceptible to voriconazole, whereas *Aspergillus* spp. were least susceptible to natamycin [13,14]. For advanced FK shown in MUTT 2, by adjunctive oral voriconazole to topical natamycin, only *Fusarium* keratitis cases may get better visual outcome [15]. Furthermore, compared to other *Fusarium* spp., *F. solani* has been shown to have higher voriconazole resistance and a worse visual outcome [16]. The evidence above suggests that the overall prognosis of FK will be increased by prompt diagnosis of FK, differentiation of *Fusarium* and *Aspergillus*, and identification of critical fungal species such as *F. solani*. 

We previously developed a dot hybridization array (DHA) for rapid diagnosis of FK, of which this assay provided much higher sensitivity than that of the culture [17]. This assay was accomplished by amplifying the internal transcribed spacer region (ITS) that contained the target gene (5.8 S rRNA gene) by polymerase chain reaction (PCR), followed by hybridization of the PCR amplicon to a fungus-specific oligonucleotide probe immobilized on a nylon membrane. It can detect fungi in the corneal scrapes within a shorter turnaround time (one working day) than that of the culture. Based on the superiority of this molecular technique, this study aimed to develop and verify a novel DHA for fulfilling the unmet clinical need by expanding its detection potential from not only diagnosing FK, but also differentiating *Fusarium* and *Aspergillus* keratitis, as well as identifying target fungal species.

## 2. Materials and Methods

### 2.1. Reference Strains and Clinical Isolates

**Figure 1:**
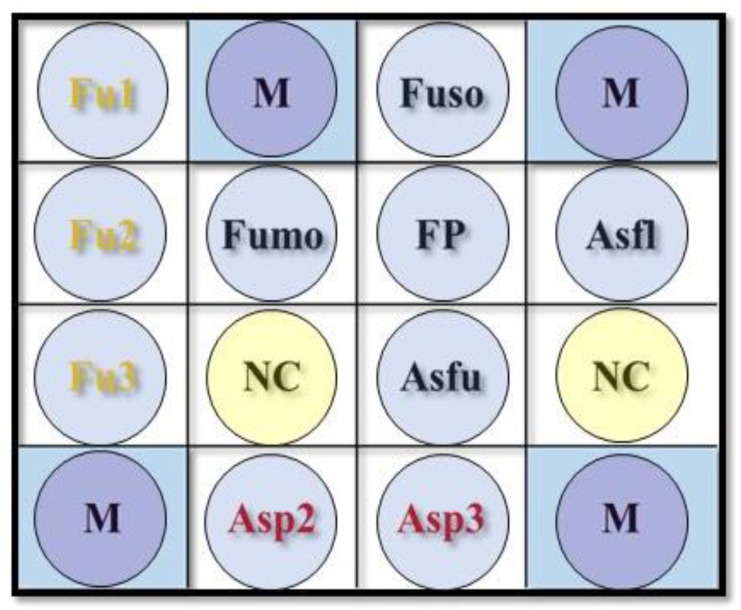
The layout of a novel dot hybridization array for detecting fungal keratitis. The universal fungal probe FP in the layout (0.6 × 0.6 cm) was designed for detecting all fungal species (Table 2). The genus probes “Fu1”, “Fu2”, and “Fu3” were designed to detect all *Fusarium* sp. The probes “Fuso” and “Fumo” were used to identify *F. solani* and *F. verticillioides*, respectively. The genus probes “Asp2” and “Asp3” were designed to detect all *Aspergillus* sp. The probes “Asfl” and “Asfu” were used to identify *A. fumigatus* and *A. flavus*, respectively. The dot “NC” is a negative control (tracking dye only). The probe “M” is a position marker, i.e., a digoxigenin-labeled oligonucleotide probe (digoxigenin-GCATATCAATAAGCGGAGGA).

Several reference strains and clinical isolates (Table 1) of fungi were used for the preclinical specificity test. A newly developed DHA (Figure 1) for detecting all fungi, *Fusarium* spp., *F. solani*, *F. verticillioides* (formerly *F. moniliforme*), *Aspergillus* spp., *A. flavus*, and *A. fumigatus* (Table 2) was developed for clinical verification via a prospective multi-center study after passing the preclinical test with target and non-target microorganisms.

### 2.2. Participants

All procedures involving human subjects adhered to the Declaration of Helsinki and were approved (approval period from 14 February 2018 to 6 February 2021) by the Committee of Medical Ethics and Human Experiments of Chang Gung Memorial Hospital (CGMH), Kaohsiung Veterans General Hospital (KVGH), and Kaohsiung Medical University Hospital (KMUH). The patients included in this study were suspected FK subjects or suspected MK subjects, of which FK could not be ruled out via clinical morphology. Patients who were less than 20 years old or more than 85 years old and unwilling to participate in this study were excluded.

### 2.3. Collection of Clinical Samples

The corneal scraping samples for the above subjects were collected from the enrolled subjects of CGMH, KVGH, and KMUH. A 15# sterilized knife was used to scrape the superficial cornea with infiltrates, especially at the margin of ulceration. One part of the corneal scrapes was sent to the section of microbiology or laboratory diagnostic department in CGMH, KVGH, and KMUH for conventional microbial examination. The remaining part was washed into a 3-mL sterile microcentrifuge tube with 2.5 mL of normal saline. The sample was then sent to our laboratory for microbial DNA extraction followed by molecular diagnostic tests. The tube was frozen in a −20 °C refrigerator up to 1 week before DNA extraction.

### 2.4. Oligonucleotide Probe Development and Fabrication of the DHA

The oligonucleotide probes were diluted 1:1 (final concentration, 10 mM) with a tracking dye solution, drawn into wells of 96-well microtiter plates, and spotted onto nylon membranes (Roche, Mannheim, Germany) as described previously [19]. Arrays were prepared with an automatic arrayer (Ezspot, Taipei, Taiwan) by using a solid pin of 400-μm diameter. A new-generation DHAs for FK was shown in Figure 1, which was designed to diagnose FK and identify two fungal genus and four fungal species via specially designed oligonucleotide probes (Table 2). The position markers were shown on the array after hybridization and helped to pinpoint the hybridized probes. After all probes had been applied, the membrane was exposed to shortwave UVs (Stratalinker 1800; Stratagene, La Jolla, CA, USA) for 30 s. For differential diagnosis, other DHAs previously established for diagnosing bacterial keratitis, herpes keratitis, acanthamoebic keratitis, and microsporidial keratitis were used on-demand [20,21,22]. 

### 2.5. DNA Extraction, PCR Amplification, and Hybridization with DNA Array

DNA was extracted using DNeasy^®^ Blood and Tissue Kit (Qiagen, Valencia, CA, USA) according to the manufacturer’s instructions with some modifications. For isolated molds, mycelium (approximately 0.5 × 0.5 cm) was acquired from the culture medium via ultrasound oscillation to the frozen tube filled with ddH_2_O. After oscillation for 30 s, we reached the mixed fluid for centrifugation. The pretreated product was obtained after the supernatant was removed. Digoxigenin (dig)-labeled ITS (internal transcribed spacer) for array hybridization was amplified by PCR using universal primers [18,19]. Each primer was labeled with a digoxigenin molecule at its 5′ end and was synthesized by Bio Basic Inc. (Markham, ON, Canada). PCR reaction mixture was prepared from the KAPA HiFi PCR Kits (Kapa Biosystems, Inc., Cape Town, South Africa). PCR thermocycling followed the condition of Bouchara et al. [23]. A negative control was performed with each run by replacing the template DNA with sterile water in the PCR mixture. The procedures were the same as those described previously [18,19], except that the hybridization step was conducted at 50 °C for 90 min.

### 2.6. Fungal DNA Sequencing for Discrepant Analysis

The gold standard for diagnosing FK in this study was (1) culture positive for fungus, or (2) DNA sequencing positive for fungus. For a cornea scraping sample that demonstrated a positive result for fungus either by culture or the DHA, the extracted DNA was re-amplified with primers ITS1/ITS5 and ITS4 (without 5′ end labeling). Then, the PCR product was used to confirm the presence of fungal DNA in the sample. The amplified ITS fragment was then sequenced, and the determined sequence was used to search for homologous sequences in GenBank using the BLASTN program (http://blast.ncbi.nlm.nih.gov; accessed on 1 September 2021).

### 2.7. Statistical Analysis

Microsoft Excel and PowerPoint 2016 (Microsoft Corporation, Redmond, WA, USA) were used as graphic tools. GraphPad Prism version 9.3.0 for Windows (GraphPad Software, San Diego, CA, USA) was used for statistical analysis. The 95% Wilson/Brown binomial confidence intervals for these indices were estimated.

## 3. Results

### 3.1. Demographic Data of Participants

**Figure 2:**
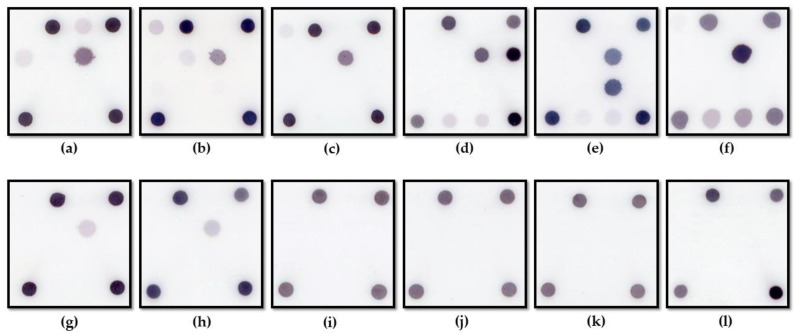
Representative results of the dot hybridization array for detecting fungal keratitis. (**a**–**c**) *Fusarium* keratitis patients with respective pathogens of *F. solani*, *F. verticillioides*, *F. delphinoides*; (**d**–**f**) *Aspergillus* keratitis patients with respective pathogens of *A. flavus*, *A. fumigatus*, and *A. niger*; (**g**,**h**) Fungal keratitis patients with respective pathogens of *Curvularia geniculata* and *Candida tropicalis*; (**i**) *Pseudomonas aeruginosa* keratitis; (**j**) herpes simplex keratitis; (**k**) *Acanthamoeba palestinensis* keratitis; (**l**) microsporidal (*Vittaforma corneae*) keratitis.

This multi-center study applied a novel fungal DHA (Figure 1 and Table 2) for simultaneously diagnosing FK, identifying two crucial fungal genera, *Fusarium* spp. and *Aspergillus* spp., and four commonly reported pathogenic fungal species, *F. solani*, *F. moniliforme*, *A. flavus*, and *A. fumigatus* (Figure 2). A total of 146 subjects, suspected FK patients or suspected MK patients, in which FK could not be ruled out via clinical manifestation, were included in this study (Table 3). The number of male subjects were significantly higher than that of the female subjects (*p* < 0.0001). There was no significant difference for the involved eye. Ocular trauma (65 patients, 44.5%) was the most common risk factor, while diabetes mellitus was the most common systemic risk factor. Among these patients, 107 (73.3%) patients were confirmed FK by culture and DNA sequencing. In addition, 39 non-FK patients, including 16 bacterial keratitis, two herpes keratitis, three microsporidial stromal keratitis, three acanthamoebic keratitis, and 15 non-infectious keratitis, were enrolled in this study.

### 3.2. Detection of Fungi in Corneal Scraping Samples

The FP probe in this fungal DHA chip was used to diagnose FK (Figure 1 and Table 2). Seven patients were fungal culture-positive but DHA-negative (Table 4). One of the seven patients was confirmed as FK by DNA sequencing, but the other six patients could not be confirmed by DNA sequencing. Twenty-seven subjects were culture-negative but DHA-positive (Table 4). According to the DNA sequencing results, 1 patient was false-positive, while the other 26 patients were true-positive. This DHA’s performance for diagnosing FK was estimated based on the diagnostic criteria of FK. The sensitivity, specificity, positive predictive rate (PPR), and negative predictive rate (NPR) were 93.5%, 97.4%, 99.0%, and 84.4%, respectively (Table 4). This result revealed that the novel DHA has good performance in the diagnosis of FK.

### 3.3. Identification of Fusarium sp. in Scrapes

Three genus probes, Fu1, Fu2, and Fu3, were used to diagnose *Fusarium* keratitis (Figure 1 and Table 2). *Fusarium* keratitis was diagnosed if any one of the three probes was positive. *F. solani* keratitis was diagnosed if the probe Fuso was positive. Similarly, *F. verticillioides* keratitis was diagnosed if the probe Fumo was positive. Based on the result of culture and DNA sequencing, the DHA’s performance in diagnosing *Fusarium* keratitis, *F. solani* keratitis, and *F. verticillioides* keratitis were estimated. The sensitivity, specificity, PPR, and NPR in diagnosing *Fusarium* keratitis were 93.2%, 93.8%, 87.2%, and 96.8%, respectively (Table 5). In addition, the sensitivity, specificity, PPR, and NPR in diagnosing *F. solani* keratitis were 83.9%, 100%, 100%, and 95.6%, respectively. The sensitivity, specificity, PPR, and NPR in diagnosing *F. verticillioides* keratitis were 100%, 99.3%, 50%, and 100%, respectively. However, only one subject had *F. verticillioides* keratitis.

### 3.4. Identification of Aspergillus sp. in Scrapes

Similarly, two genus probes, Asp2 and Asp3, were used to diagnose *Aspergillus* keratitis (Figure 1 and Table 2). *Aspergillus* keratitis was diagnosed if any one of the two probes was positive. A. flavus keratitis was diagnosed if the probe Asfla was positive. Similarly, *A. fumigatus* keratitis was diagnosed if the probe Asfum was positive. Similar to *Fusarium* keratitis, the DHA’s performance in diagnosing *Aspergillus* keratitis, *A. flavus* keratitis, and *A. fumigatus* keratitis were respectively estimated. The sensitivity, specificity, PPR, and NPR in diagnosing *Aspergillus* keratitis were 83.3%, 100%, 100%, and 99.3%, respectively (Table 6). Moreover, the accuracy in diagnosing both *A. flavus* keratitis and *A. fumigatus* keratitis was 100%. However, there were only two patients with *A. flavus* keratitis and two patients with *A. fumigatus* keratitis.

## 4. Discussion

FK is acknowledged as a catastrophic MK with a slow yet relentless clinical course. It had a changeable presentation during the progression of corneal infection. FK should be differentiated from herpetic and acanthamoebic keratitis in the early epithelitis dominant phase, while it should be distinguished from bacterial, necrotizing herpetic, and microsporidial stromal keratitis in the late stromal infiltration stage. Moreover, FK carried a higher failure rate of medical treatment than other MK, especially for patients with delayed diagnosis and erratic application of corticosteroids. *Fusarium* and *Aspergillus* are two major genera that cause FK. However, *Fusarium* spp. is more susceptible to natamycin than to voriconazole, whereas *Aspergillus* spp. is more sensitive to voriconazole than to natamycin [14,24,25]. Accordingly, the treatment outcome via topical voriconazole was inferior to that via natamycin for a FK cohort with a higher prevalence of *Fusarium* keratitis [26], and *Aspergillus* keratitis had a higher medical failure and surgical rate under natamycin treatment [27]. The novel DHA developed in this study had an excellent diagnostic performance in diagnosing FK. Moreover, it was capable of differentiating between *Fusarium* and *Aspergillus* keratitis and recognizing *F. solani* keratitis. Therefore, our DHA is helpful in providing an earlier diagnosis and the opportunity for a more precise treatment for FK.

DHA is a highly sensitive technique with the potential to develop an oligonucleotide array for identifying fungal pathogens to species level [18]. We previously applied this technique to diagnose FK [17] and assessed the bacterial bioburden for the orthokeratology lens care system [28]. However, FK has broad spectra of fungal pathogens, which are almost opportunistic by means of ocular trauma. A universal probe for detecting fungus is not enough to help physicians in determining a personalized anti-fungal strategy. In this study, the novel DHA showed promising results for this goal because it is capable of diagnosing FK and differentiating between *Fusarium* and *Aspergillus* keratitis. 

Among seven patients with culture-positive but DHA-negative for fungi (Table 4), only one patient was confirmed by DNA sequencing. The possible cause of failed DHA detection for this patient was that simultaneous detection of several targets may have dispersed DNA amplicons to different probes, which increased the detection limit of the universal probe and would have needed more microorganisms in a scrapping sample. The other six patients could not be confirmed by DNA sequencing for fungi. We speculated that the result was caused by sampling failure, which led to no or insufficient microorganisms in the scrape for the DHA assessment. Among the 27 patients with culture negative but DHA positive (Table 4), 26 patients were confirmed positive but 1 patient was false-positive by discrepant analysis. DHA was more sensitive than culture, which cannot detect fastidious or nonviable microorganisms [17]. However, DHA is a susceptible molecular test, which may detect very few contaminated fungi or fungal amplicons and cause a false-positive result.

Too few *F. verticillioides* keratitis led to failure to estimate sensitivity and PPR, and therefore, we could only conclude that probe Fumo had reasonable specificity and NPR (Table 5). Thus, the DHA’s performance for diagnosing *F. verticillioides* keratitis could not be sufficiently verified. However, the DHA’s performance was well-validated for diagnosing *Fusarium* keratitis and *F. solani* with high accuracies of 93.6% and 96.4%, respectively. Among the three false-negative scrapes via probes Fu1, Fu2, and Fu3 for detecting *Fusarium* spp., two samples with *F. solani* and one sample with *Fusarium* spp. were confirmed by DNA sequencing. Amplicons distributed to the universal probe FP and the species probe Fuso could be the false-negative reason. Among the six false-positive scrapes via probe Fuso for detecting *F. solani*, four samples were recognized as *Colletotrichum* spp. (two *C. siamense*, one *C. fructicola*, and one *C. gloeosporioides*), one sample was identified as *Scedosporium apiospermum*, and one sample was confirmed as *Gjaerumia* spp. One sample was a false-positive detection for *F. verticillioides*, where the DNA sequencing result was *F. solani*. Modifying the species’ probes Fuso and Fumo for specific detection of *F. solani* and *F. verticillioides*, or designing new probes for *Colletotrichum*, *Scedosporium*, and *Gjaerumia* will be considered.

There were only six scrapes with *Aspergillus* spp., including two *A. flavus* keratitis, two *A. fumigatus* keratitis, one *A. niger*, and one *A. tamarii*. Therefore, it was weak to estimate sensitivity and PPR for the probes for diagnosing *Aspergillus genera*, *A. flavus*, and *A. fumigatus* (Table 6). Only reasonable specificity and NPR could be claimed for these probes. For the sample with false-negative detection for *Aspergillus* spp. (by probes Asp2 and Asp3), the result of DNA sequencing was *A. tamarii.* The design of a new probe for detecting *A. tamarii* or the modification of current genus probes will be the solution for avoiding misdiagnosis for the species.

Due to the limitation in which some target pathogens were rare, the sensitivity and PPR of the probes for identifying *Aspergillus* keratitis and the species probes for recognizing *F. verticillioides*, *A. flavus*, and *A. fumigatus* could not be confidently validated. However, these probes undoubtedly had acceptable specificity and NPR. Moreover, they were speculated to have a similar performance to the probes for diagnosing *Fusarium* keratitis and *F. solani* keratitis because all probes had passed the preclinical challenge with target and non-target microorganisms (Table 1).

According to the comprehensive review of Hoffman et al. [11], filamentous FK, particularly *Fusarium* and *Aspergillus* keratitis, is a global treat. FK has an apparent geographical variation, and *Fusarium* or *Aspergillus* keratitis often accounted for the top two prevalent pathogens, even in North America. In some temperate areas such as Europe and North America, *Candida* spp. has a chance to be in the top two most common pathogens of FK. The severity of *Candida* keratitis is less than that of filamentous FK. Thus, the DHA can also be used in non-Asian countries to detect *Candida* spp. via probe FP and exclude vision-threatening *Fusarium* and *Aspergillus* keratitis via species and genus probes. The DHA can be used as an adjunctive clinical test of conventional culture for a mycology laboratory to increase the recovery rate and efficiency in diagnosing FK and differentiating *Fusarium* from *Aspergillus* keratitis for early precise anti-fungal treatment. However, the procedures need well-trained staff and sterile technique in the laboratory.

There are several novel antifungal regimens for FK [29,30,31]. Keratosept ophthalmic solution containing hexamidine diisethionate 0.05% is a potential candidate for the treatment of *Candida* and staphylococcal infections of the ocular surface [29]. Corneal collagen cross-linking treatment via Riboflavin/UVA (CXL) showed therapeutic efficacy against FK [30]. CXL combined with 0.02% chlorhexidine is also an effective therapy against FK, particularly for multi-resistant *Fusarium* keratitis [31]. Following the development of novel anti-fungal treatments, we believe the DHA, or a modified DHA targeting specific fungal species or genus, can provide a rapid and precise diagnosis helping physicians to choose a suitable anti-fungal treatment.

## 5. Conclusions

The novel DHA had an excellent diagnostic performance in diagnosing FK, differentiating *Fusarium* and *Aspergillus* keratitis, and identifying *F. solani* keratitis. Although, its performance in diagnosing *Aspergillus* spp., *A. flavus*, *A. fumigatus*, and *F. verticillioides* was not well-verified in this study due to limited cases, this DHA revealed a promising result toward rapid diagnostic precision medicine. We believe this DHA will improve the prognosis of FK via early diagnosis and more precise guidance of anti-fungal regimens.

## Figures and Tables

**Table 1:** Reference strains and clinical isolates for preclinical test of the fungal dot hybridization assay.

Species	Reference Strain (s) ^a^	No. of Clinical Isolates	Total No. of Strains
Target fungal species for sensitivity test for species and genus probes			
*Fusarium solani*	ATCC 36031, CBS 109028, BCRC 32448	6	9
*Fusarium verticillioides*	BCRC 31492, BCRC 31745, BCRC 35113, BCRC 32878	0	4
*Fusarium oxysporum*	ATCC 26225, CBS 798.95	0	2
*Other Fusarium* spp.	BCRC33554	4	5
*Aspergillus flavus*	BCRC 30006, BCRC 30007, BCRC 30008, BCRC 30009, BCRC 30187	2	7
*Aspergillus fumigatus*	BCRC 30099, BCRC 30502, BCRC 32120, BCRC 32149, BCRC 32836	1	6
*Aspergillus niger*	BCRC 30201, BCRC 30204, BCRC 31130	0	3
*Aspergillus nidulans*	ATCC 11267, ATCC 13833, BCRC 30100	0	3
*Aspergillus terreus*	BCRC 30135, BCRC 31128, BCRC 32068	0	3
*Aspergillus clavatus*	BCRC 31116, BCRC 31486, BCRC 31736	0	3
*Aspergillus versicolor*	BCRC 30225, BCRC 31123, BCRC 31488	0	3
Non-target fungal species for specificity test for species and genus probes			
*Curvularia* spp.	CBS 351.65, BCRC 30899, CBS 102694, CBS 149.71, CBS 148.63	2	7
*Candida albicans*	BCRC 20511, BCRC 20512, BCRC 20513	0	3
*Candida krusei*	BCRC 20514, BCRC 21321, BCRC 21720	0	3
*Candida glabrata*	BCRC 20586, CBS 860, CBS 861	0	3
*Candida parapsilosis*	BCRC 20515, BCRC 21253, BCRC 21544	0	3
*Candida tropicalis*	BCRC 20520, BCRC 21436, BCRC 21560	0	3
*Candida guilliermondii*	BCRC 20862, BCRC 21549, BCRC 21500	0	3
*Candida rugosa*	BCRC 21356, BCRC 21709	0	2
*Acremonium* spp.	BCRC 33315, BCRC 32239	1	3
*Bipolaris* spp.	CBS 274.52	1	2
*Pseudallescheria boydii*	ATCC 44329, ATCC 44331, ATCC 44332	0	3
*Cryptococcus neoformans*	BCRC 20528, BCRC 20532, BCRC 22873	0	5
Non-target species from non-fungal pathogens for specificity test for all probes			
*Staphylococcus aureus*	BCRC 10451, BCRC 15287	0	2
*Staphylococcus epidermidis*	BCRC 10785, BCRC 15245	0	2
*Streptococcus pneumoniae*	BCRC 14733, BCRC 10794	0	2
*Acinetobacter baumannii*	BCRC 10591, BCRC 15884	0	3
*Moraxella catarhalis*	BCRC 10629, BCRC 10628	0	2
*Klebsiella pneumoniae*	BCRC 11644, CCUG 15938	0	4
*Escherichia coli*	BCRC 15481, BCRC 15484	0	4
*Pseudomonas aeruginosa*	BCRC 10944, ATCC 27853	6	8
*Serratia marcescens*	BCRC 15326, BCRC 11576	0	5
*Burkholderia cepacia*	BCRC 13208, BCRC 13906	0	2
*Stenotrophomonas maltophilia*	BCRC 10737	0	3
*Mycobacterium chelonae*	ATCC 35749, CCUG 37827	0	2
*Mycobacterium fortuitum*	BCRC 15320, JCM 6387	0	2
*Mycobacterium abscessus*	NCTC 10269	0	1
Herpes simplex virus type 1		2	2
Herpes simplex virus type 2		2	2
Varicella zoster virus	Rod strain	3	4
*Encephalitozoon cuniculi*	ATCC 50789	0	1
*Encephalitozoon hellem*	ATCC 50504	0	1
*Encephalitozoon intestinalis*	ATCC 50651	0	1
*Vittaforma corneae*		1	1
*Acanthamoeba castellanii*	ATCC 30010, ATCC 50374, ATCC 50370	0	3
*Acanthamoeba griffini*	ATCC 30731, ATCC 50702	0	2

^a^ ATCC, American Type Culture Collection, Manassas, Va., USA; CBS, Centraalbureau voor Schimmelcultures, Utrech, The Netherlands; BCRC: Bioresources Collection and Research Center, Hsinchu, Taiwan; CCUG, Culture Collection, University of Göteborg, Sweden; JCM: Japan Collection of Microorganisms, RIKEN BioResource Research Center, Ibaraki, Japan; NCTC: National Collection of Type Cultures, Central Public Laboratory Service, London, UK.

**Table 2:** Probes used in the dot hybridization array.

Target Microorganism	Probe Code ^a^	Sequence (5′ to 3′)	Length (bp)	Tm ^b^ (°C)	Location	GenBank Accession No.
All fungi	FP [17]	GCATCGATGAAGAACGCAGCttttttttt ^c^	20	57.2	228–247	FR727118
*Fusarium solani*	Fuso [18]	AGTAGCTAACACCTCGCGACTGGAGA	26	56.0	446–471	AF129105
*Fusarium verticillioides*	Fumo [18]	CGAGTCAAATCGCGTTCCCCAAATTG	26	54.4	395–420	AY533376
*Aspergillus flavus*	Asfl [18]	CGAACGCAAATCAATCTTTTTCCAGGT	27	51.6	512–538	AY373848
*Aspergillus fumigatus*	Asfu [18]	GCCAGCCGACACCCAACTTTATTTTTCTAA	30	55.2	213–242	AY230140
*Fusarium* sp.	Fu1 ^d^	GCGTCATTTCAACCCTCAAGCCCC	24	63.7	340–363	AM412639
	Fu2 ^d^	CTTCTGAGTAAAACAAGCAAATAAAT	26	48.9	164–189	AM412639
	Fu3 ^d^	AGCTTCCATAGCGTAGTAGYAA	22	53.8	442–463	AM412639
*Aspergillus* sp.	Asp2 ^d^	GGACGGGCCCRAAAGGCAGCGGCGGC	26	77.8	426–451	AF138290
	Asp3 ^d^	GGCAGCGGCGGCACCGYGTCCGGTCCT	27	79.7	440–466	AF138290

^a^ Oligonucleotide probes are arranged on the dot hybridization array, as indicated in Figure 1. ^b^ Tm = melting temperature. ^c^ Multiple bases of thymine (t) were added to the 3′ end of the probe. ^d^ Newly designed probes used in this study.

**Table 3:** Demographic data of subjects.

Clinical Parameters	Value
Number of patients	146
Age (years; mean ± s.d.)	59.3 ± 15.8
Sex (women/men; no./no.)	52/94
Disease eye (OD/OS; no./no.)	72/74
Final diagnosis (no.)	
Fungal keratitis	107
Bacterial keratitis	16
Herpes keratitis	2
Acanthamoebic keratitis	3
Microsporidial stromal keratitis	3
Noninfectious keratitis	15
Major risk factors (no.)	
Trauma	65
Contact lens wear	14
Dirty water exposure	7
Ocular surface disease	3
Neurotrophic keratopathy	1
Lagophthalmos	3
Facial palsy	1
Hyperthyroidism	1
Diabetes mellitus	11
Chemotherapy	1
Undetermined	39

**Table 4:** The performance of the dot hybridization array for diagnosing fungal keratitis.

*N* = 146		Culture		Culture or DNA Sequencing		Sensitivity	Specificity	PPR	NPR
		Positive	Negative	Positive	Negative	(C.I.; %)	(C.I.; %)	(C.I.; %)	(C.I.; %)
DHA	Positive	74	27	100	1	93.5	97.4	99.0	84.4
	Negative	7	38	7	38	(87.1–96.8)	(86.8–99.9)	(94.6–100.0)	(71.2–92.3)

DHA = dot hybridization array; PPR = positive predictive rate; NPR = negative predictive rate; C.I. = confidence interval.

**Table 5:** Diagnostic performance of *Fusarium* keratitis after discrepant analysis.

*N* = 140 ^a^			Post-Discrepancy		Sensitivity	Specificity	PPR	NPR
			Positive	Negative	(C.I.; %)	(C.I.; %)	(C.I.; %)	(C.I.; %)
DHA	*Fusarium* sp.	Positive	41	6	93.2	93.8	87.2	96.8
		Negative	3	90	(81.8–97.7)	(87.0–97.1)	(74.8–94.0)	(90.9–99.1)
	*F. solani*	Positive	26	0	83.9	100.0	100.0	95.6
		Negative	5	109	(67.4–92.9)	(96.6–100.0)	(87.1–100.0)	(90.1–98.1)
	*F. verticillioides*	Positive	1	1	100.0	99.3	50.0	100.0
		Negative	0	138	(5.1–100.0)	(96.0–100.0)	(2.6–97.4)	(97.3–100.0)

^a^ Diagnostic performance was estimated after excluding six cases with suspected sampling failure (positive culture but negative DNA sequencing results). DHA = dot hybridization array; PPR = positive predictive rate; NPR = negative predictive rate; C.I. = confidence interval.

**Table 6:** Diagnostic performance of *Aspergillus* keratitis after discrepant analysis.

*N* = 140 ^a^			Post-Discrepancy		Sensitivity	Specificity	PPR	NPR
			Positive	Negative	(C.I.; %)	(C.I.; %)	(C.I.; %)	(C.I.; %)
DHA	*Aspergillus* sp.	Positive	5	0	83.3	100.0	100.0	99.3
		Negative	1	134	(43.7–99.2)	(97.2–100.0)	(56.6–100.0)	(95.9–100.0)
	*A. flavus*	Positive	2	0	100.0	100.0	100.0	100.0
		Negative	0	138	(17.8–100.0)	(97.3–100.0)	(17.8–100.0)	(97.3–100.0)
	*A. fumigatus*	Positive	2	0	100.0	100.0	100.0	100.0
		Negative	0	138	(17.8–100.0)	(97.3–100.0)	(17.8–100.0)	(97.3–100.0)

^a^ Diagnostic performance was estimated after excluding six cases with suspected sampling failure (positive culture but negative DNA sequencing results). DHA = dot hybridization array; PPR = positive predictive rate; NPR = negative predictive rate; C.I. = confidence interval.

## Data Availability

Data is fully available upon reasonable request to corresponding author.
